# Dentistry in the Orient

**Published:** 1897-02

**Authors:** Richard Henry Kimball

**Affiliations:** Chicago, Ill.


THE
AMERICAN JOURNAL
—OF—
DENTAL SCIENCE.

VOL. XXX. BALTIMORE, FEBRUARY, 1897. NO. 10.

ARTICLE I.

### DENTISTRY IN THE ORIENT.

BY RICHARD HENRY KIMBALL, D. D. S., CHICAGO, ILL.

Read before the Chicago Dental Society.

Under this caption I purpose making the attempt to introduce you to a few of the scenes and events of a dentist's life in China. Some will interest you purely from a professional standpoint; at the same time I must crave your indulgence, if, as we wander about, there are introduced many incidents that the most charitable interpretation would fail to make relative to dentistry.

Recently, when your committee requested me to prepare a paper for the June meeting of the Society upon "Dentistry in China," I asked to be assigned to a later meeting in the year, as it was my desire that, before attempting to present this or any subject akin to it, I should succeed in obtaining certain dates, figures, facts and other matter, which I am getting together, most of which relates to this particular subject.

I felt that, with the native's crude outfit and a few exhibitions of his handicraft before you, augmented by a series of lantern slides illustrating various interesting features of Chinese life, I might perhaps succeed in presenting in an acceptable, and possibly entertaining manner, what I happened to know concerning "Dentistry in China." How then, with all of the just mentioned entertaining features lacking, and the very short time at my disposal, am I to cope with the greatly broadened subject given in the list of papers for the year, of "Dentistry in the Orient."

The title is certainly comprehensive, and perhaps even misleading, since I fear it may lead you to expect more than it is possible for me to provide at this time.

Geographically the subject extends far beyond the limits of my personal knowledge, and I shall in the main attempt to deal with it only so far as I can speak from individual experience and observation. In treating any subject relating to the Orient, it is not only important but essential that we have some knowledge of the history of the particular country under consideration. It is not my intention to enter upon any historical sketch of China, for probably none of you are particularly interested in the earlier or even the later history of this people, it is nevertheless true that they are, as has been so often stated "peculiar." They are much more than that; they are a wonderful people, and present quite a curiosity in the history of nations, a careful study of which gives the student a valuable and important clew to the character of the Chinese people.

We are apt to forget, even if we ever fully realized the fact, that the bit of strange humanity that occasionally drifts near us, in the living whirlpool of our city streets, is a citizen of what is unquestionably the oldest nation in the world—as has been said—"their history dates back to a period where no historian would dare to state an exact date."

They speak the same language, and observe the same social customs they did several thousand years before the Christian era, and they are the only living people to-day who were contemporaneous with the Egyptians, Assyrians and the Jews.

They have carried on their own affairs and struggles; maintaining almost unchanged the boundaries and proportions of their empire, ignorant and indifferent concerning the events taking place about them, and which make up the history of the other nations of Europe.

As early as A. D. 1295, Marco Polo had traveled extensively through China, and brought to Europe the first knowledge of that other country lying still further to the East—Japan.

It is some three and a half centuries since foreigners (Europeans) settled at Macao, near the mouth of the Canton river; and they have enjoyed regular intercourse with the Chinese for all this period, with no perceptible impression on China. It is inconceivable, the small effect the other nations have until recently exercised upon her; indeed it is only within the present century that this influence can be said to have made any impression at all. The reason for this is largely in the fact that the Chinese do very little traveling even in their own country, generations following each other for ages in the same town. The customs and dialect become local, and the people of one province can not converse with those of the next province, although possibly not fifty miles intervene. The written language is the same throughout the Empire; but if we consider the many different dialects, and the closeness with which each separate community clings to its own habitation, the difficulty of spreading any influence is readily understood.

During my sojourn in the "East" I was naturally desirous to obtain all the information I could concerning the progress of the Chinese in that field of science in which I was particularly interested, but I was unable from lack of time to extend my research much beyond the limits of foreign residence. Careful observation whenever opportunity presented, and inquiry of those resident among them, failed to reveal even the most primitive attempt at preserva tion of the natural teeth. I, therefore, believe that I am fully within the bounds of truth when I say that beyond certain remedies for relieving toothache, extracting and an occasional crude attempt to supply an artificial tooth, or a few teeth, there is no knowledge whatever of dentistry among the hundreds of millions of Chinese.

In the treaty ports with the daily contact with foreigners there has naturally grown up in the minds of many of them some knowledge of the medical and dental methods of the western world, and from the medical missionary. Many even of those living in the far interior, have come to appreciate the value and importance of foreign medical and surgical skill.

None of them are so ignorant of dental matters, however, that they cannot appreciate, in some degree at least, the distress and inconvenience of an aching tooth, and employ some agency for relief, it may be spiritual, medical or mechanical. It is the quite generally accepted idea with them that pain in a tooth is caused by the presence of a "worm" which has taken up its abode inside of it.

They are encouraged in this belief by the class of men who go about the city streets extracting teeth. These find it to their pecuniary advantage to cater to this notion, and to prove the truth of their assertion, it is their frequent habit after extracting a tooth where the imprisoned "worm" has been making things particularly lively, to break the tooth and exhibit the "worm" to the astonished victim and interested onlookers. I wish I could bring before you a picture of an aspirant for dental honors, as I first saw him at a little village a few miles out from Hong Kong. He was squatting by the roadside, in front of one of the shops on the main street, proclaiming in loud tones his skill as a "worm" and tooth extractor. (This I learned from a Pigeon English speaking native.) His outfit consisted of a wooden tray some two feet long, fifteen inches wide and perhaps three inches deep, elevated upon a stool. At either end of the tray an upright was fastened, and between these several wires extended upon which were strung hundreds of teeth. In the tray were perhaps enough more teeth to fill a peck measure, besides a number of bottles and paper packages.

I enlisted the aid of the friendly interpreter to find out about the bottles and ascertain what appliances he was using for extracting, there being no instrument whatever in sight.

He was shyness itself, but after much palaver he produced from that part of his clothing made famous by Bret Harte a rather large pair of ancient, much worn and abominable dirty pliers, of foreign manufacture. These, after some hesitation, he permitted me to take for examination, and I was conscious as I held them in my hand of a profound feeling of respect for the simple tool, the product of some humble European artisan, probably long dead, who in making it had toiled to a better purpose than he had ever dreamed.

In all probability either you or I would have discarded the implement as not retaining enough of its original usefulness to be of service in tack pulling, and, but for all the horribly abundant septic possibilities present, would have doubted that this was the instrument used, and according to his statement, the only one.

No amount of questioning elicited the least information regarding the other articles in his tray (the bottles and packages), a shake of the head being the only response to my repeated inquiries. Common report tells of a mysterious powder some of these men used in extracting teeth, a small amount of which is placed on the gum around the tooth to be extracted, when after waiting several minutes the tooth can be removed with the fingers.

After much difficulty and several years of waiting I finally succeeded in obtaining, through a Chinese acquaintance, what was said to be a specimen of this powder, and its analysis shows it to be composed of potassium nitrate, sodium sulphide and what seems to be red sealing wax. That only the fingers are used in removing the tooth is true, the powder being employed simply to mystify, which it succeeds in doing thoroughly, deceiving many foreigners as well as Chinese, for I have frequently been assured by the former that the powder did the work. A wonderful degree of strength is developed in fingers by long years of practice in pulling wooden pegs from boards. In Japan the boys are put to this exercise when quite young, and as a result pegs are easily withdrawn by them that we would find difficult to remove with forceps.

As an object lesson in Chinese dentistry I coveted this outfit, and offered the man what was a large sum for it; but he refused to sell. Then it was proposed to pay him his own price, including a good pair of forceps, but to no purpose; he could not be induced to part with anything. It may not be amiss to speak, in passing, of the peculiar trait of the Chinese illustrated in this incident. It is characteristic of them that no matter how much they may wish to dispose of an article, how desperately they may be in need of the money its sale will bring, they invariably refuse to sell when openly approached with an offer to buy.

They have their customary way of conducting such matters, and with them custom outweighs in importance every other consideration, almost to that of life itself.

The preliminaries to a sale must be conducted with great discretion, for an axiom of much importance in China is, "The country villager is born perverse; the more you wish to buy the more he is determined not to sell."

Suspicion is a national characteristic; not as applied to one's neighbor solely, but each man suspects himself, or recognizes his own weakness, and in all trades, except in shops and open market, where prices are fixed, the employment of an intermediary is necessary. I cannot dwell longer on this trait than to quote the Chinese adage, "I

there are no clouds in the sky there will be no rain on the earth; if there is no one to stand between, business will not be done."

For years I have been trying to get possession of the outfit of one of these Nomadic individuals, even enlisting the assistance of residents of interior cities, where I felt that the prejudical conditions might not be so great, but thus far without success. I am hopeful, however, that the day may come when I shall make that addition to my collection, and have the pleasure of exhibiting it to you. What I have stated is, so far as I have been able to ascertain, the sum total of the Chinaman's own unaided efforts on behalf of the natural teeth.

They make an indifferent attempt at supplying the places of lost teeth, the artificial being made of ivory or bone, shaped with a file and attached to the remaining teeth with brass wire. The effect in the mouth is often hideous beyond description. Where a single tooth is lost, an artificial substitute is shaped to fit the vacancy and forced to place, no retaining wire being used. When the space has enlarged until that piece is no longer retained, another and wider piece is made that will fit tight. This wedging operation is repeated until the retaining teeth assume such an angle that it becomes necessary to wire the piece in place. I have with me some specimens of this class of work, and appliances with which they were made, which you may be interested in examining.

The file, with its teeth cut only one way, is a very fair tool you will observe, rather coarse for such work, but it is marvelous how much they accomplish with just such instruments. It is of native construction and its temper is poor.

The drill is made from an umbrella rib, while the wire is doubtless of foreign manufacture.

In some of the larger coast cities are to be found a few Chinese practicing dentistry. "Alle same foreign dentist," they would tell you. Their knowledge of things dental is purely empirical, and has been derived from association with foreign dentists; either as assistant or an attendant, whose duties were to receive visitors and keep the office in order.

During his tenure of office he had probably permitted his sponge-like proclivities to operate with some freedom, and so had "absorbed" enough of the worn, or nearly worn out belongings of his master, to fit him out very fairly when he decided to set up for himself.

Two of these men I know to be possessed of a considerable degree of skill; one, practicing in Hong Kong, was for several years Doctor Rogers' assistant, and when I first went there in 1886, was patronized by a number of the leading foreigners in preference to the only European dentist there at that time. The other, who is now practicing in Shanghai, was assistant to Doctor H. H. Winn for fifteen or more years before I joined him, and continued with us until the time of Doctor Winn's death in 1890, thus enjoying a pupilage of fully twenty years.

Both of these men have fully equipped modern dental offices, furnished in one case with a Wilkerson high low-base chair and fountain spittoon, and in the other with an S. S. White pedal lever chair. Each has a full equipment of White's instruments. All of these Chinese practitioners are patronized quite largely by a class of foreigners whose means will not admit of their going to the foreign dentist, such as merchant and naval seamen, soldiers (in Hong Kong) and many others. They are also doing a good work in educating their own people to some knowledge of dental matters, as many of the better class of Chinese go to them for both operative and prosthetic work. As each one of these native "tooth doctors," as they are called, has a small army of relatives and family connections who, like parasites, attach themselves to him, their value as educators is greatly increased, and I predict that it will not be many years before the foreign dentist in China wlll have Chinese graduates from American dental colleges to compete with.

A large and gradually increasing number of Chinese patronize the foreign practitioner. They are the higher officials and wealthy merchants who constitute a very satisfactory class of patients in many respects, but I must admit that I prefer not to work for them and did nothing to encourage their coming to me.

The directions in which they are satisfactory are, the absence of nerve and the ready promptness with which they unquestionably pay their bills. Nerves, as we know them in the occident, do not exist in the Chinese. I never knew one to make the slightest movement while in the chair, no matter what the severity of the operation. The first experience I had with this nervelessness made a decided impression upon me. One morning during the early weeks of my China life, the assistant came in with the announcement that Mr.——was there, naming a Chinese gentleman, whom the records showed to have been a patient for several years. I gave instructions to have him shown in, and there filed in a splendidly attired Chinaman followed by three Chinese women, each bearing what appeared at first sight to be another smaller woman upon her back; when the burdens were deposited in chairs they proved to be daughters of my visitor, ranges in ages, I judged, from nine to twelve years and carried, because their bound feet would not admit of their walking.

In perfect English I was requested to examine the mouths of the children and do whatever seemed to be necessary. Each in turn was carried to the operating room and from one to three temporary teeth removed from each mouth.

There was not a second's delay waiting for a mouth to be opened, not an audible sound, not a tear from either one of the girls from beginning to end of the operations. Nerves! Apparently there were none. Rev. Arthur H. Smith, in writing of "The Absence of Nerves," in his "Chinese Characteristics," says, "One of the most perfect exemplifications of the automatic nature of Chinese physical activity with which we are acquainted, is the process of malleting for a dentist. Those who have been compelled to submit to this form of torture, know how difficult it is in an occidental land to get a person to mallet, who shall deliver his strokes in an even succession, and of a uniform weight. It takes long practice upon a long line of victims, before anything like a steady average is maintained. Now watch the nearly automatic operation of the "boy" in the office of the first dentist toward which harsh fate drives you in China. The boy is a very nearly ideal machine, and he never knows that he is using his nervous system at all, as perhaps indeed he is not."

I am glad to add my testimony to the superior excellence of the Chinese assistant; as a malleter, perfect, as a faithful, attentive helper, unsurpassed. Always at his post, ready and quick to learn. If he does annex any of your old belongings, which is not unlikely, you do not like censuring him; it is done so gracefully and at such an opportune time, that you feel almost under obligations to him for relieving you of the necessity of disposing of the articles. My experience differs from that of many touching their honesty, for, during the years my "boy" was in my service I can not recall an instance of an article being missing that was left in his care.

I have spoken of one assistant who was in the office for a period of twenty years; when I left China I turned over to my successor as a part of my office belongings my "boy" (the head servant in office or house is known by that name regardless of the fact that he may be a grandfather), who entered my service eight years before, also the patriarch of my servant staff, the office coolie, who then was transferred to the fourth generation of dentists in that office, he being over thirty years in one situation, or what was virtually the same employ.

Since my return to America I have frequently been asked in various ways, but with perfect seriousness, this question: "Are you not glad to be back again where you can enjoy the comforts of civilization and have respectable patients to work for?" The frequency of this inquiry leads me to believe that there is a very widely mistaken idea prevailing in the minds of many relative to this, and also to other than dental matters, in the far east; and that it may not be uninteresting if I introduce some description of the everyday life of the foreigner in Hong Kong, Shanghai or Yokohama. I particularize these places because it is only here that dentists reside, and what is true there is in a large msasure true of the other cities where foreigners are living.

"Respectable patients to work for" and "comforts of civilization!" One would suppose that we worked for coolies, lived in miserable quarters and were obliged to subsist on a diet of cats and dogs, to mention nothing worse. In what I have said I have given you some idea of the proportion of Chinese in my practice. It was small, but I presume quite as large as that enjoyed by my associates. The Chinaman we meet upon the streets of our cities is no more to be compared to the Chinese Mandarin or rich merchant in his costly apparel of silk and satin, than is the laborer shoveling dirt on the railroad nearby to be compared to the gentleman that comes to the office of any one here. Such a Chinaman as I describe is a respectable, polite gentleman always; nor does he bring with him nerves enough for ten people, or beset you with a never ending flood of objections to this, that and the other thing, as too often is the case with our patrons in our more civilized America. The clientele of the leading dentists in China would compare favorably with that of the best in any country on the globe; nobility, titled diplomats of European courts, ministerial, consular and judicial representatives of all the principal nations, these, and their families, the merchant princes from Europe and America, with their families, the officers from the American and European men of war on the station, and hundreds of English speaking missionaries constitute a field of practice most delightful in its personality, and one that any dentist could feel honored in possessing.

With the exception of the missionaries, the foreigners in China are there either in Chinese government employ, in official relations with the government; or in trade, and reside only at the open, or treaty ports, and Peking. These cities are hundreds of miles apart and communication is only by water.

Their houses are large, airy two-story structures, built of brick, surrounded at both stories and on as many sides as possible with broad verandas.

Shanghai furnishes one of the most abundant markets in the world—one with which the most fastidious epicureans could find no fault.

To supply the dental requirements of China and Japan there are ten foreign dentists; those Chinese I have described; and a large number of native dentists in Japan.

There are two American dentists in Japan, both in Yokohama. The conditions for practicing in Japan are less favorable than in China, owing to the more intelligent competition of the natives, some of whom are graduates of our schools of dentistry.

The eight dentists who occupy the China field are graduates of American dental colleges and all but one are Americans, three of whom are in Hong Kong, in two offices, and the remaining five in Shanghai, operating three offices.

When a foreigner in one of the smaller ports finds he can no longer postpone a visit to the dentist, he tells his "boy" to pack the luggage and they go to Shanghai, or Hong Kong, as the case may be for a week or fortnight. Sometimes the dentist does the traveling, going to the other coast ports, the Yangtsze River ports, or perhaps to Peking, for a month. On such trips he is the guest of some one at each port, setting up his chair where he can; often his host's dining room serves as office. Occasionally patients come great distances to consult you; one lady traveled two thousand miles, being four weeks on the trip, traveling afoot, horseback and in boat and steamer.

Without more than the usual amount of self-reliance, the dentist will not succeed in these remote points, for there is no dental supply house at his elbow to fall back upon, no society meetings for mutual help and suggestions; he must alone grasp every problem that is presented and solve it at any cost.

On one occasion while in Hong Kong a Chinese official came to me, sent by a medical friend in Canton; his teeth were all right, but he had lost his left ear and wanted to know if I could do something for him, because, while so disfigured, it was impossible for him to enter the presence of his superiors. I was able to put him in presentable form and intended photographing him. His delight when he saw the restored ear was so great, however, that he rushed for the Canton boat not waiting for the paint on the new ear to become dry, and that was the last I ever saw of him. Another case—that I failed with —was when a Mandarin at Soochow, some eighty miles from Shanghai, sent down by a friend requesting that I make him "plates for both jaws," as his teeth were entirely gone. It is necessary to have a very extensive outfit for practicing in China; every instrument must be in duplicate to provide against breakage; gold plate and foil by the ounce, thousands of artificial teeth and quantities of every other article must be kept in stock. Even then, without the greatest care, something will give out, with the dental depot three months distant.

In my effort to give you some conception of the practice of dentistry in China, I have said nothing of the severe climate, the great heat during part of the summer and other features connected with life in that country. There is much more that could be said, but I will close with this word of advice to the younger men, don't decide at once that China is just the field you have been looking for and pack your little case of instruments and start for "Far Cathay." If you have friends there, or can take a pocket full of letters of introduction it perhaps might be thought of; lacking them you could but make a dismal failure of the venture, for the field is fully occupied, and furthermore, the people are not of the changing kind.—*The Dental Review.*

